# Heavy on Plans, Light on Delivery: The Structural Failures of Ethiopia's Nutrition Policies

**DOI:** 10.1111/mcn.70073

**Published:** 2025-08-04

**Authors:** Taddese Zerfu

**Affiliations:** ^1^ International Food Policy Research Institute (IFPRI) Addis Ababa Ethiopia

## Abstract

Ethiopia's development ambitions rest on the foundation of a healthy population, yet its nutrition sector remains stalled despite decades of planning and investment. Nearly 38% of children under five are stunted, and food insecurity continues to affect millions. Landmark initiatives like the National Food and Nutrition Policy and the Seqota Declaration demonstrate strong political will—but implementation and scale‐up falters due to entrenched structural failures. At the core of this breakdown is an overstretched and under‐resourced frontline workforce. Health Extension Workers, while committed, are burdened with wide‐ranging responsibilities, and lack the specialized training needed for effective nutrition service delivery. As a result, national strategies often collapse at the community level, where change is most urgently needed. This is further compounded by fragmented coordination. Despite the multisectoral nature of malnutrition—spanning health, agriculture, education, and social protection—ministries and partners frequently work in silos, sending conflicting messages to the same households. Meanwhile, valuable research and data remain disconnected from policy and program implementation, limiting the system's responsiveness and accountability. The path forward requires more than incremental fixes. Ethiopia needs specialized community nutrition workers to bridge the last‐mile gap, a high‐level coordination mechanism to align sectoral actions, and agile policies grounded in real‐time evidence. Without these structural reforms, the burden of malnutrition will continue to erode the country's human capital and economic potential. This is not just a health crisis—it is a critical bottleneck to national progress. The time for structural transformation is now.

## Summary

- Despite strong national plans and political commitments, Ethiopia's nutrition policies often collapse at the community level due to structural failures.
- An overstretched and under‐resourced frontline workforce—the Health Extension Workers—limits effective nutrition service delivery at the community level, where real intervention happens.
- Fragmented multisectoral coordination and the disconnect between research and program implementation hinder coherent action and policy responsiveness.

## Background

Ethiopia stands at a pivotal moment. As a low‐income nation aspiring to attain middle‐income status, its economic ambitions rest on a critical foundation: a well‐nourished, healthy population. Yet despite a proliferation of nutrition policies, strategies, and interventions over the past decades, the country continues to grapple with some of the highest rates of child undernutrition globally. Nearly 38% of children under five are stunted (EPHI and ICF 2019), and chronic food insecurity affects roughly one in five households (FAO, IFAD, UNICEF, WFP and WHO).

The paradox is stark. Ethiopia is not lacking in ambition or policy frameworks. Landmark initiatives such as the National Food and Nutrition Policy (2018) (MoH—Ethiopia 2018) and the Seqota Declaration (Government of Ethiopia 2025)—its flagship commitment to end child stunting by 2030—reflect strong political intent. The nutrition landscape is also crowded with actors, including multiple ministries, international NGOs, and research institutions. Yet progress remains frustratingly slow. Stunting rates are declining by only 0.5%–1% annually, well below the pace required to meet national and global nutrition targets (Government of Ethiopia 2025).

## Why Ethiopia's Nutrition Sector Resembles a ‘Broken‐Leg’ Animal?

The metaphor of a ‘broken‐leg’ animal perfectly captures the state of Ethiopia's nutrition sector—an entity that has all the necessary limbs but can barely move forward. Just as an injured animal struggles despite its natural capabilities, Ethiopia's nutrition system is hobbled by structural weaknesses that prevent progress. The issue is not a lack of policy vision, political will, or active stakeholders. Rather, the real obstacle lies in the failure to implement nutrition strategies effectively, especially at the community level where they matter most. The system is injured where it needs to be strongest: at the interface between national plans and local delivery.

### Policies Without Proper Implementers

At the heart of Ethiopia's nutrition delivery system is the Health Extension Program, which depends extensively on Health Extension Workers (HEWs) to translate national nutrition policies into concrete actions at the community level (Assefa et al. 2019; Workie and Ramana 2013). These frontline workers are tasked with delivering services ranging from child growth monitoring and dietary counselling to supplement distribution and household‐level behaviour change interventions (Assefa et al. 2019). In principle, they serve as the linchpin of Ethiopia's nutrition system. Globally, community health workers (CHWs) play an equally central role. As highlighted by Pallas et al (Pallas et al. 2013), they are essential in bridging the persistent gap between political commitment and real‐world health implementation, particularly in low‐resource settings where formal health infrastructure is often inadequate.

In practice, however, they are being stretched far beyond capacity (Assefa et al. 2019; Zerfu et al. 2023; Gobezie et al. 2023; Haileamlak and Ataro 2023). Each HEW often serves a population of 3000 to 5000 people, making it nearly impossible to provide individualized attention or sustained support to vulnerable households. Nutrition is just one of many priorities on their overloaded agenda, which also includes maternal and child health, immunizations, sanitation, communicable disease control and more.

To make matters worse, most HEWs are not equipped with specialized knowledge in nutrition (Workie and Ramana 2013). They may know how to distribute micronutrient powders or recognize visible signs of malnutrition, but deeper work—such as promoting sustained dietary change, counselling on infant and young child feeding, or facilitating multisectoral referrals—often lie beyond both their training and formal scope of work. This reflects a broader challenge observed globally: the scaling up of CHW programs has frequently outpaced investments in their training, supervision, and support systems (Perry et al. 2021). As a result, policies that appear comprehensive on paper falter during implementation, not due to lack of ambition, but because the individuals tasked with executing them are often unsupported, undertrained and overburdened.

### Fragmented Efforts and Weak Coordination

Another core reason the nutrition sector limps along is the lack of effective coordination among key stakeholders (Kennedy et al. 2016; Mersha et al. 2025). Ethiopia has rightly adopted a multisectoral approach to nutrition, acknowledging that food security, hygiene, health services and education all shape nutritional outcomes (MoH—Ethiopia 2018). But in practice, the institutions tasked with carrying out these responsibilities often work in isolation.

Multiple ministries—including Health, Agriculture, Education, and Women and Social Affairs—have mandates related to nutrition (Kennedy et al. 2016, 2015, 2020). Additionally, NGOs, UN agencies, and donors operate various programs across the country. Yet instead of a synchronized system, what exists is a patchwork of parallel efforts. These actors often develop their own plans, use different reporting systems, and send conflicting messages to the communities they serve (Linnet 2012).

A clear example of this fragmentation can be seen at the household level: one family might be advised by agricultural extension agents to grow high‐value cash crops, while health workers encourage them to focus on subsistence farming and dietary diversity. Without harmonized messaging, shared monitoring tools, and joint planning mechanisms, families are left confused—and opportunities for impact are diluted.

This problem is neither unique to Ethiopia nor new. As Sanghvi et al. (2025) emphasize, two decades of implementation research on complementary feeding show that without structural reforms to align goals, financing, and accountability across sectors, multisectoral nutrition efforts often stall at the operational level (Global Nutrition Report: Action on Equity to End Malnutrition 2020). Similarly, Pérez‐Escamilla and Engmann (Pérez‐Escamilla and Engmann 2019) argue that integrating nutrition into broader systems requires deliberate political commitment, institutional capacity, and incentives for collaboration across ministries and partners. Ultimately, the lack of aligned incentives and mechanisms for cross‐sectoral collaboration undermines the very promise of a multisectoral approach, stalling the comprehensive effort needed to reduce malnutrition at scale.

### Research That Fails to Translate Into Action

Ethiopia is not short of nutrition‐related research (Federal Ministry of Health FMOH & Ethiopian Public Health Institute EPHI 2021). Universities, public health institutes, and development partners regularly publish studies exploring the causes and consequences of undernutrition, as well as evaluating the effectiveness of various interventions. Topics range from complementary feeding (Ahmed et al. 2020; Gemede et al. 2025; Berhanu et al. 2019) and micronutrient deficiencies (Herrador et al. 2014; Hassen et al. 2020) to the potential of school feeding programs and biofortified crops.

Yet, the critical bridge between knowledge generation and policy action is weak (Walls et al. 2018). Research findings are rarely translated into practical program designs or policy reforms (Morankar et al. 2024; Tangcharoensathien et al. 2022; Kaba and Kitaw 2018). Even when there is clear evidence supporting effective interventions—such as community‐based management of acute malnutrition (CMAM) or locally produced fortified foods—these solutions are not scaled up in a systematic way. Institutional inertia, lack of interministerial buy‐in, insufficient funding, and limited dissemination channels all contribute to this gap (Morankar et al. 2024).

The result is a paradox: while knowledge accumulates in academic journals and donor reports, the frontline delivery systems remain unchanged. Policymakers and implementers are often unaware of, or unable to act on, the insights generated by research, leaving promising innovations stranded in pilot projects or small‐scale initiatives (Morankar et al. 2024; Kaba and Kitaw 2018).

## What Must Change? Pathways Toward a Functional Nutrition System in Ethiopia

Escaping the ‘broken leg’ trap in Ethiopia's nutrition sector will require more than marginal adjustments. It demands bold, systemic reforms grounded in a genuine systems approach—one that matches the scope and complexity of the challenge at hand (Chitekwe et al. 2024). While Ethiopia has no shortage of vision and commitment, the way forward must focus on making the system functional—especially at the community level—by investing in people, coordination, and data‐driven decision‐making.

### Building a Dedicated Nutrition Workforce at the Community Level

One of the most urgent reforms is the creation of a dedicated grassroots workforce that can focus exclusively on nutrition. The current model, which relies almost entirely on HEWs, is not sustainable. These workers are overwhelmed with a wide range of responsibilities and cannot be expected to deliver high‐impact nutrition interventions on top of everything else (Zerfu et al. 2023; Haileamlak and Ataro 2023).

To address this gap, Ethiopia must consider training and deploying a new cadre of community‐based nutrition workers. These individuals would work in tandem with HEWs but would be tasked specifically with stunting prevention, dietary counselling, household‐level behaviour change, and child feeding practices. Unlike the generalist HEWs, the new grassroot nutrition experts would bring focused expertise and continuity to nutrition programming at the household and community levels.

### Enforcing Stronger and Smarter Multi‐Sectoral Coordination

Malnutrition is driven by interconnected challenges in health, agriculture, WASH, education, and gender (Workman et al. 2022; Reinhardt and Fanzo 2014). Tackling it requires strong, multisectoral coordination—yet Ethiopia's efforts remain fragmented. A high‐level National Nutrition Delivery Unit could align ministries, coordinate partners, and enforce accountability through shared targets and joint monitoring.

Nutrition must also be embedded into agriculture. Farmers should be supported to grow diverse, nutrient‐rich crops, with extension services promoting nutrition‐sensitive goals to improve household diets and translate food production into real nutritional gains.

### Embracing Data‐Driven, Adaptive Policies

If Ethiopia is to make meaningful progress, its nutrition policies must become more responsive to real‐time data and grounded in evidence of what works. Rigid, top‐down programming must give way to adaptive, feedback‐informed interventions that can evolve as local needs change. Ethiopia should invest more in implementation research (Brown et al. 2021). While academic studies abound, there is a lack of operational insights into how to make nutrition programs work effectively within Ethiopia's complex, decentralized governance, and resource‐constrained environment. Embedding learning loops into nutrition programming—testing interventions at scale, measuring their impact, and rapidly refining them—will be essential for sustained progress (Brown et al. 2021; Tumilowicz et al. 2019).

## Conclusion: A Make‐Or‐Break Moment for Ethiopia's Future

Ethiopia's malnutrition crisis threatens its development ambitions. Despite strong policies, progress stalls due to fragmented implementation and overburdened and poorly trained grassroot implementers—the HEWs. Breaking this cycle requires urgent action: dedicated community nutrition workers, true multisectoral coordination, and research‐driven solutions. Without bold reforms, the costs—lost potential, economic drag, and entrenched poverty—will mount. Fixing this ‘broken leg’ is not just about better nutrition— it is about unlocking Ethiopia's future. The time for transformative action is now.

## Conflicts of Interest

The author declares no conflicts of interest.
